# A multicenter study of the distribution pattern of posterior corneal astigmatism in Chinese myopic patients having corneal refractive surgery

**DOI:** 10.1038/s41598-020-73195-w

**Published:** 2020-09-30

**Authors:** Yijun Hu, Shanqing Zhu, Lu Xiong, Xuejun Fang, Jia Liu, Jin Zhou, Fangfang Li, Qingsong Zhang, Na Huang, Xiaohua Lei, Li Jiang, Zheng Wang

**Affiliations:** 1 Aier Institute of Refractive Surgery, Refractive Surgery Center, Guangzhou Aier Eye Hospital, Guangzhou, China; 2 Aier School of Ophthalmology, Central South University, Fourth Floor, New Century Mansion, 198 Middle Furong Road, Changsha, 410015 China; 3 Refractive Surgery Center, Shenyang Aier Eye Hospital, Shenyang, China; 4 Refractive Surgery Center, Chengdu Aier Eye Hospital, Chengdu, China; 5 Refractive Surgery Center, Wuhan Aier Eye Hospital, Wuhan, China; 6 Refractive Surgery Center, Hankou Aier Eye Hospital, Wuhan, China

**Keywords:** Diagnostic markers, Diagnostic markers

## Abstract

Including posterior corneal astigmatism (PCA) into consideration may increase the accuracy of astigmatism correction after corneal refractive surgery. In the present study we aim to investigate the distribution pattern of PCA in a large number of myopic patients from multiple ophthalmic centers. There were 7829 eyes retrospectively included in the study. Pentacam data of the eyes were retrieved from the machine and only results with image quality labelled with ‘OK’ were included. Distribution of PCA was slightly positively skewed (Skewness = 0.419, Kurtosis = 0.435, KS *P* < 0.0001). Mean PCA was 0.34 ± 0.14 D (range: 0.00 D-0.99 D). PCA was ≥ 0.25 D in 74.91% of the eyes and was ≥ 0.50 D in 11.61% of the eyes. In 97.55% of the eyes the steep meridian of PCA was vertical (SMV). PCA magnitude was significantly higher in eyes with SMV PCA (*P* < 0.0001) or high manifest astigmatism (MA, *P* < 0.0001). There was a significant correlation between anterior corneal astigmatism (ACA) magnitude and PCA magnitude in all of the eyes (*r* = 0.704, *P* < 0.0001). There was also a trend of decreasing frequency and magnitude of SMV PCA with aging (both *P* < 0.0001). In conclusion, PCA is present in myopic patients having corneal refractive surgery and PCA magnitude is increased with higher MA or ACA. Consideration of the impact of PCA on laser astigmatism correction may be necessary.

## Introduction

Ocular astigmatism is one of the major factors affecting postoperative visual function after corneal refractive surgery^1^. With the increasing demand of the patients and surgeons for better postoperative visual quality, proper management of the ocular astigmatism has become increasingly important for corneal refractive surgery. Major components of the ocular astigmatism include the anterior corneal astigmatism (ACA), the posterior corneal astigmatism (PCA), and the astigmatism induced by the crystalline lens^2^. In corneal laser surgery such as laser in situ keratomileusis (LASIK), correction of corneal astigmatism is often performed based on the ACA^3^. This method is not entirely unproblematic if the patients have a significant ocular residual astigmatism (ORA) of which PCA is one of the major components^4^. It was reported that the efficacy of LASIK in eyes with a low ORA was twice as good as in eyes with a high ORA^5^. It was also shown that the ORA was strongly correlated with the PCA after LASIK surgery (*r* = 0.81)^6^. Thus, it is of clinical importance to investigate the PCA distribution in patients having corneal refractive surgery.

Another scenario where PCA may need to be taken into consideration is astigmatism correction by cataract surgery. Accurate evaluation of preoperative total corneal astigmatism (TCA) is crucial for precise astigmatism correction in patients undergoing cataract surgery^7^. Traditional methods of calculating TCA were mainly based on the ACA, known as keratometric astigmatism, assuming the cornea a single dioptric surface with a fixed anterior to posterior curvature ratio^8^. However, increasing evidences have suggested that this approach may lead to inaccurate estimation of the TCA due to neglecting the PCA^9–12^. On the contrary, including PCA into calculation of TCA increases the accuracy of astigmatism correction^13,14^.

There have been several reports about PCA pattern in patients having assessment for refractive surgery or in patients about the same age^9,15,16^. However, results from these reports may not be directly applied to our patients since there may be significant differences in posterior corneal metrics among different ethnic groups^17^. Nevertheless, to date there is still no information about PCA distribution pattern in Chinese myopic adults, which is the largest population of refractive surgery candidates in the world. In the present study, we aim to investigate the distribution pattern of PCA in Chinese myopic patients having corneal refractive surgery by pooling together the data from multiple ophthalmic centers.

## Results

The patients were from five ophthalmic centers, including Guangzhou Aier Eye Hospital (GZ), Shenyang Aier Eye Hospital (SY), Chengdu Aier Eye Hospital (CD), Wuhan Aier Eye Hospital (WH), and Hankou Aier Eye Hospital (HK). There were 7829 patients (7829 eyes) included in the study and 57.01% of them were male. Mean age of the patients was 25.1 ± 5.4 years. Mean spherical equivalent (SE) of the eyes was − 4.87 ± 1.66 D. There was significant difference in age, gender, SE and PCA magnitude among patients from different ophthalmic centers (all *P* < 0.0001). Demographics of the eyes are shown in Table 1.

**Table 1:** Demographics of the subjects in different ophthalmic centers.

	GZ	SY	CD	WH	HK	*P*^†^
Eyes (N)	2227	2434	1470	1431	267	N/A
Eyes (%)	28.45%	31.09%	18.78%	18.28%	3.41%	N/A
Male (%)	47.55%	63.60%	63.27%	51.85%	68.91%	< 0.0001
Age (year)^a^	26.9 ± 5.5	23.9 ± 5.1	24.1 ± 5.5	25.4 ± 5.1	23.6 ± 4.7	< 0.0001
SE (D)^a^	− 4.78 ± 1.63	− 4.84 ± 1.70	− 4.92 ± 1.64	− 5.01 ± 1.58	− 5.00 ± 2.08	< 0.0001
PCA (D)^a^	0.33 ± 0.14	0.36 ± 0.13	0.31 ± 0.14	0.32 ± 0.12	0.34 ± 0.13	< 0.0001

*N* number of eyes, *SE* spherical equivalent, *PCA* posterior corneal astigmatism, *D* diopter, *GZ* Guangzhou Aier Eye Hospital, *SY* Shenyang Aier Eye Hospital, *CD* Chengdu Aier Eye Hospital, *WH* Wuhan Aier Eye Hospital, *HK* Hankou Aier Eye Hospital.

^a^Presented as mean ± standard deviation.

^†^*P* value for comparison among the five groups using Kruskal–Wallis test.

**Figure 1:**
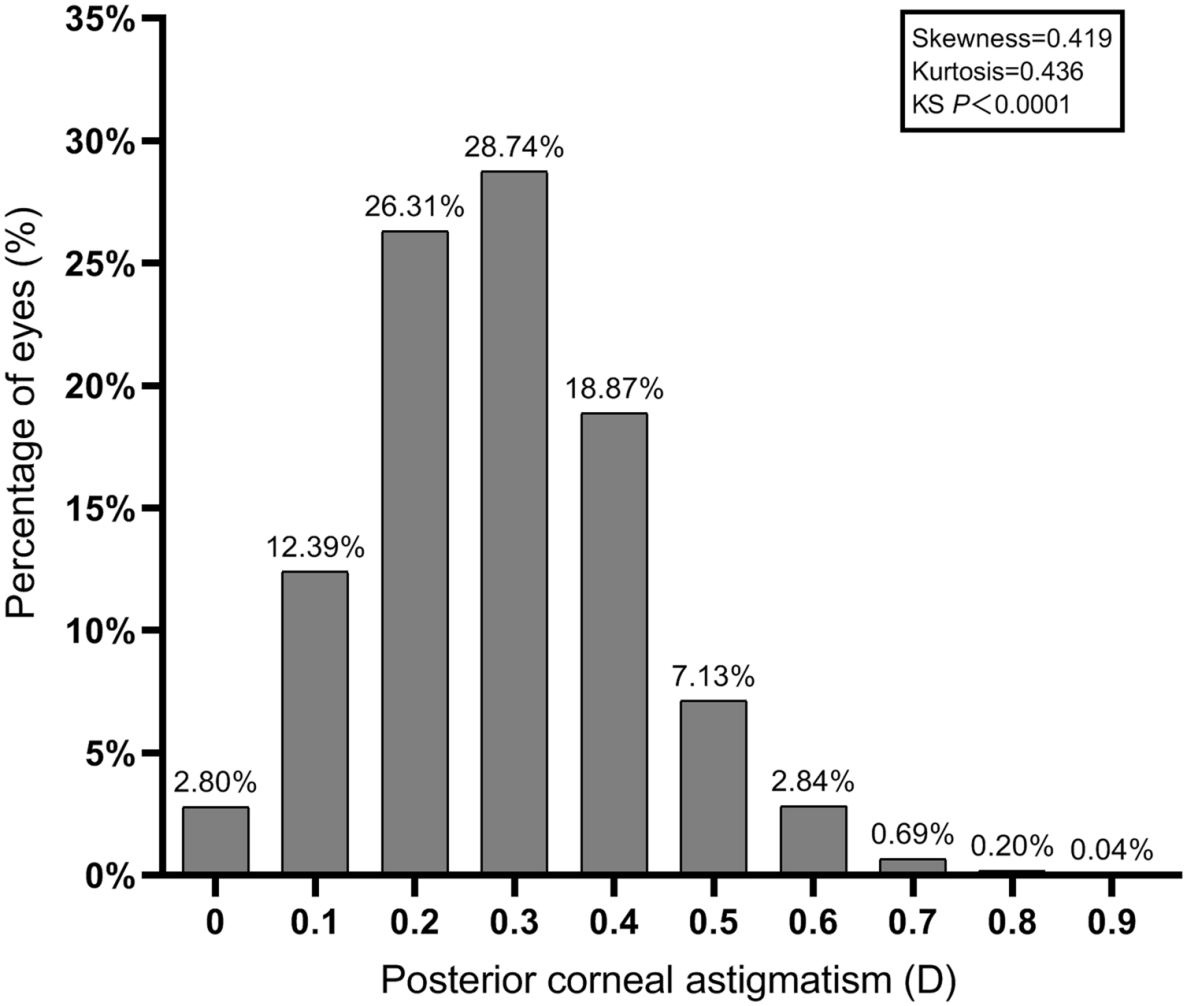
Frequency distribution of posterior corneal astigmatism.

Distribution of PCA was slightly positively skewed (Fig. 1; Skewness = 0.419, Kurtosis = 0.435, KS *P* < 0.0001). Mean PCA was 0.34 ± 0.14 D (range: 0.00 D-0.99 D) with a 95% confidence interval of 0.33 D-0.34 D. Aggregate PCA was 0.10 ± 0.36 @ 1.53. PCA was ≥ 0.10 D in 97.47% of the eyes, ≥ 0.25 D in 74.91% of the eyes and ≥ 0.50 D in 11.61% of the eyes.

Details about the PCA in different groups of eyes are shown in Table 2. In 97.55% of the eyes the PCA’s axis was steep meridian vertical (SMV), 0.65% of the eyes was steep meridian horizontal (SMH), and 1.80% of the eyes was steep meridian oblique (SMO). PCA magnitude was 0.34 ± 0.13 D for SMV, 0.10 ± 0.09 D for SMH and 0.15 ± 0.10 D for SMO. PCA magnitude of SMV was significantly higher than SMH and SMO (both *P* < 0.0001). Aggregate PCA was 0.01 ± 0.37 @ 1.56 for SMV, 0.03 ± 0.14 @ 179.65 for SMH, and 0.02 ± 0.18 @ 90.28 for SMO. PCA magnitude was 0.32 ± 0.13 D in eyes with low myopia, 0.33 ± 0.13 D in moderate myopia, and 0.36 ± 0.14 D in high myopia. PCA magnitude was statistically significantly higher in eyes with high myopia compared to low and moderate myopia, but the difference was negligible (both *P* < 0.0001). Aggregate PCA was 0.01 ± 0.35 @ 183.65 for low myopia, 0.01 ± 0.36 @ 0.84 for moderate myopia, and 0.01 ± 0.38 @ 3.19 for high myopia. PCA magnitude was also higher in eyes with a manifest astigmatism (MA) ≥ 2.00 D compared to MA < 2.00 D (all *P* < 0.0001).

**Table 2:** Posterior corneal astigmatism in different groups of patients.

Groups	N (%)	Mean (D)	SD (D)	Aggregate astigmatism
SMV	7637 (97.55%)	0.34*	0.13	0.01 ± 0.37 @ 1.56
SMH	51 (0.65%)	0.10	0.09	0.03 ± 0.14 @ 179.65
SMO	141 (1.80%)	0.15	0.10	0.02 ± 0.18 @ 90.28
Low myopia	987 (12.60%)	0.32	0.13	0.01 ± 0.35 @ 183.65
Moderate myopia	4744 (60.60%)	0.33	0.13	0.01 ± 0.36 @ 0.84
High myopia	2098 (26.80%)	0.36^†^	0.14	0.01 ± 0.38 @ 3.19
MA < 0.50 D	2646 (33.80%)	0.29^‡^	0.11	0.01 ± 0.31 @ 0.62
0.50 D ≤ MA < 1.00 D	2991 (38.20%)	0.32	0.12	0.01 ± 0.34 @ 0.09
1.00 D ≤ MA < 2.00 D	1875 (23.95%)	0.40	0.14	0.01 ± 0.42 @ 0.45
2.00 D ≤ MA < 3.00 D	275 (3.51%)	0.51	0.14	0.06 ± 0.53 @ − 0.99
MA ≥ 3.00 D	42 (0.54%)	0.61	0.16	0.07 ± 0.64 @ 177.19

*N* number of eyes, *D* diopter, *SD* standard deviation, *SMV* steep meridian vertical, *SMH* steep meridian horizontal, *SMO* steep meridian oblique, *MA* manifest astigmatism.

*P < 0.0001 for comparison of SMV to SMH or SMO.

^†^*P* < 0.0001 for comparison of high myopia to low myopia or moderate myopia.

^‡^*P* < 0.0001 for comparison between any two of the MA groups, expect for comparison between the groups of 2.00 D ≤ MA < 3.00 D and MA ≥ 3.00 D.

**Figure 2:**
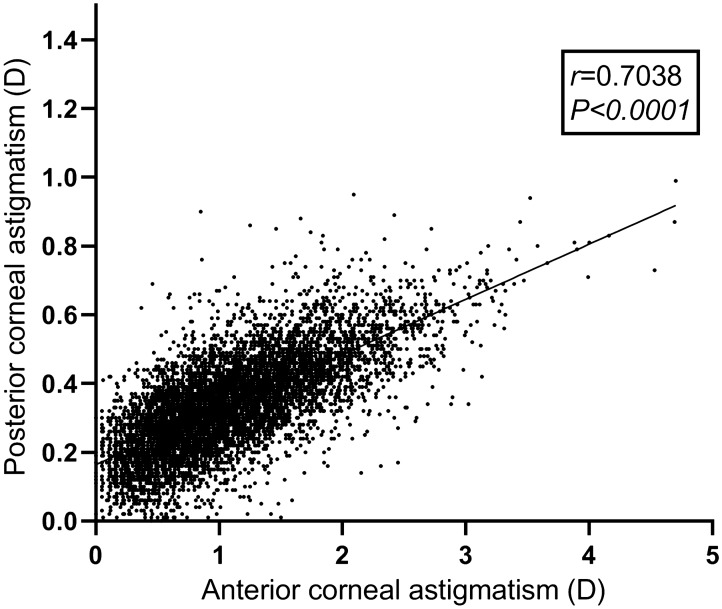
Scattergram showing correlation between anterior corneal astigmatism magnitude and posterior corneal astigmatism magnitude in all of the eyes.

There was a significant correlation between ACA magnitude and PCA magnitude in all of the eyes (Fig. 2; *r* = 0.704, *P* < 0.0001). When the ACA was with-the-rule (WTR, *r* = 0.682, *P* < 0.0001) or oblique astigmatism (OBL, *r* = 0.276, *P* < 0.0001), the correlations were positive. The correlation was negative when the ACA was against-the-rule (ATR, *r* = − 0.268, *P* < 0.0001). However, in eyes with the same magnitude of ACA, the range of PCA magnitude was wide. For example, for a WTR ACA of 1.00 D the PCA could be 0.19–0.67 D.

**Figure 3:**
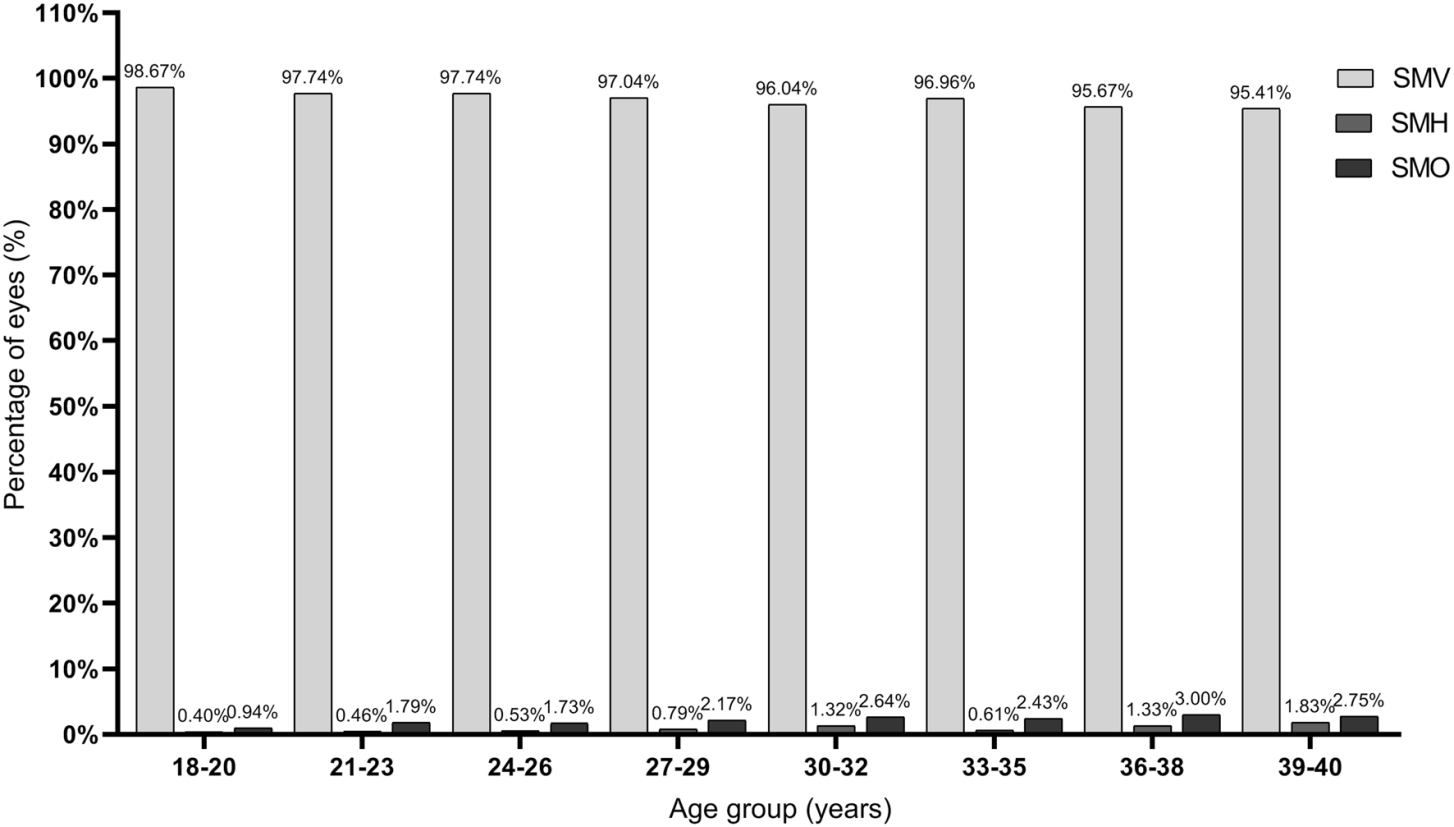
Frequency distribution of posterior corneal astigmatism in different age groups. *SMV* steep meridian vertical, *SMH* steep meridian horizontal, *SMO* steep meridian oblique.

**Figure 4:**
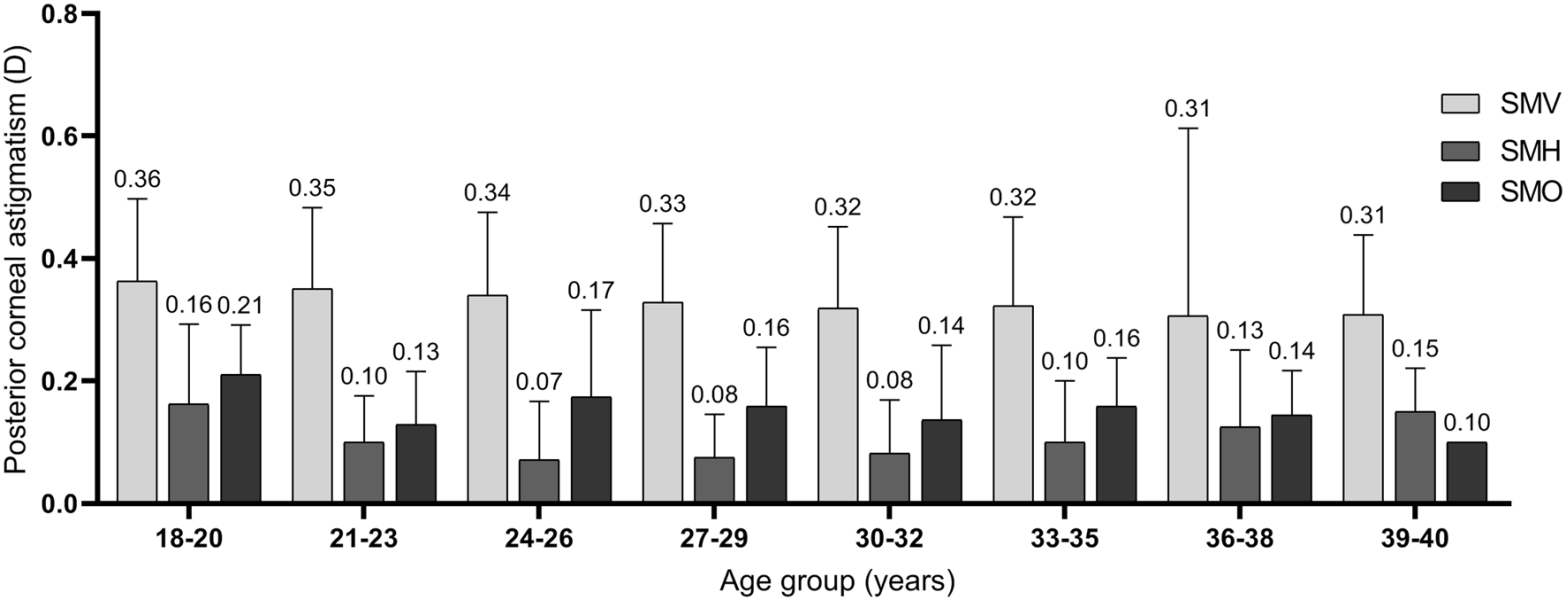
Mean magnitude of posterior corneal astigmatism in different age groups. *SMV* steep meridian vertical, *SMH* steep meridian horizontal, *SMO* steep meridian oblique.

There was a weak correlation between PCA magnitude and asphericity index of the posterior corneal surface in all of the eyes (*r* = − 0.158, *P* < 0.0001). The correlation was statistically significant only when the PCA was SMV (*r* = -0.147, *P* < 0.0001). There was a trend of decreasing frequency and magnitude of SMV PCA with aging (Figs. 3 and 4; both *P* < 0.0001).

## Discussion

In the present multicenter study, we demonstrated the distribution pattern of PCA in myopic patients from different parts of mainland China (GZ from the south, SY from the northeast, CD from the southwest, WH and HK from the central). Using the pooled data of the five cohorts with diversity in age, sex distribution and SE, we found that the PCA was ≥ 0.10 D in 97.47% of eyes, and ≥ 0.50 D in 11.61% of eyes. The PCA was SMV in 97.55% of eyes and PCA magnitude was increased in eyes with higher MA. There was a significant correlation between ACA magnitude and PCA magnitude in all of the eyes although eyes with the same ACA magnitude might have a wide range of PCA magnitude.

With the development of technology and higher anticipation for visual outcomes, accurate astigmatism correction is a hot topic in corneal refractive surgery. The ORA, an ocular astigmatism known as the discrepancy between manifest astigmatism and ACA^18^, may be associated with efficacy of the corneal refractive surgery^5^. In a previous study, it was shown that the efficacy of astigmatic correction by LASIK was significantly higher in eyes with low preoperative ORA, and conventional LASIK was twice as efficacious in the low-ORA group as in the high-ORA group^5^. In another study, it was also demonstrated that each diopter of preoperative ORA reduced the efficacy of LASIK surgery by 0.07^19^. Although in phakic eyes lenticular astigmatism is the major cause of ORA, the contribution of the PCA can’t be neglected in some cases^20^. It is noteworthy that PCA is mainly responsible for the ORA in pseudophakic eyes^21^, and it is important to consider the impact of the PCA when planning corneal refractive surgery in these eyes (such as enhancement after cataract surgery). Therefore, exploring the details of the distribution pattern of PCA in patients having corneal refractive surgery may help the surgeons better plan the treatment profiles. China has the largest number of refractive surgery candidates in the world and the PCA distribution pattern of these patients are urgently warranted. Although there have been reports about PCA in Chinese cataract patients and myopic children, to date the details of PCA distribution pattern in myopic Chinese adults have not been investigated.

Besides corneal refractive surgery, another clinical scenario in which astigmatism calculation is of remarkable importance is cataract surgery. Calculation of TCA has become a hot topic in correction of preexisting corneal astigmatism during cataract surgery. Traditional methods of estimating TCA based on ACA alone have been shown to cause significant errors in clinical practice and including PCA into the formula could increase the accuracy of astigmatism correction^9–14^. Koch et al. reported that PCA exceeded 0.50 D in 9% of eyes and anterior corneal measurements underestimated TCA by 0.22 @ 180 and exceeded 0.50 D in 5% of eyes^9^. Savini et al. also found that keratometric astigmatism overestimated WTR astigmatism by 0.22 ± 0.32 D and oblique astigmatism by 0.13 ± 0.37 D, and underestimated ATR astigmatism by 0.21 ± 0.26 D^10^. Piñero et al. observed a trend of overestimation by 0.19 D in WTR astigmatism and underestimation by 0.29 D in ATR astigmatism using keratometric astigmatism to calculate TCA^12^. Collectively, these findings suggest that including PCA into consideration is crucial to obtain an accurate TCA calculation. It is also suggested that in cataract surgery with toric IOL implantation, nomograms for PCA estimation yield superior results compared to the direct measurements of the PCA^22,23^.

In the present multicenter study, we found a mean PCA of 0.34 ± 0.14 D with a range of 0.00 D-0.99 D in Chinese myopic patients having corneal refractive surgery. The result was consistent with previous studies showing a mean PCA of 0.24 D-0.37 D in healthy eyes from subjects of the similar ages^9,15,16,24^. In our study, PCA ≥ 0.50 D was found in 11.61% of the eyes. In previous studies, a wide range of eyes with PCA ≥ 0.50 D has been demonstrated. Koch et al. reported that PCA exceeded 0.50 D in 9.0% of eyes with cataract or refractive error^9^ and Feizi et al. observed a 7.8% of eyes with PCA ≥ 0.50 D in patients having refraction assessment^16^. On the contrary, Savini et al. showed that PCA exceeded 0.50 D in 55.4% of eyes with moderate to high astigmatism^10^. In the meantime, previous studies also showed that PCA could partially compensate ACA to a range of 13.4–31.0%^25,26^. Taking the results of our study and the previous studies together, we may conclude that a substantial number of myopic patients have a non-negligible PCA, and the impact of PCA on the accuracy of astigmatism correction by corneal refractive surgery is needed to be considered in these patients.

In our study, the PCA was SMV in 97.55% of eyes and magnitude of SMV PCA was significantly higher than SMH PCA and SMO PCA. These findings were consistent with a commonly accepted theory that the PCA could partially compensate the ACA^25,26^, since in most of the young adults the ACA was WTR^9,11,16^. Besides, in our study there were positive correlations between the ACA and PCA magnitudes when the ACA was WTR or OBL, and there was a negative correlation between magnitudes of ATR ACA and PCA. Significant correlations between magnitudes of ACA and PCA were also reported in previous studies^9–11,16^. Our results also showed that eyes with a higher MA also had higher PCA. These results indicate that the compensating effects of PCA are dependent on the magnitude of ACA and MA. Therefore, in patients with higher ACA or MA, including the PCA into consideration is getting more crucial for planning a corneal refractive surgery with better astigmatism correction. It was suggested that in eyes with WTR ACA > 2.0 D or ATR ACA > 1.8 D, consideration of the PCA would lead to more accurate astigmatism correction^27^.

A wide range of PCA magnitude was observed in eyes with the same magnitude of ACA in our study. For example, the range of PCA was 0.01–0.67 D when the WTR ACA was 1.0 D. Koch et al. also found that in eyes with a WTR ACA of 1.0 D, the PCA ranged from less than 0.1 D to more than 0.5 D^9^. Variation of PCA magnitude in eyes with the same ACA magnitude was also observed in other studies^10,11,16^. These findings suggest that the PCA may not be accurately estimated using linear correlation or regression models based on ACA alone. Direct measurement of the posterior cornea seems to be a more reliable way to obtain an accurate PCA.

Our study demonstrated a trend of decreasing frequency and magnitude of SMV PCA in patients in older age groups. Similar results were also reported in previous studies^9,11,16^. Considering the shift of ACA from WTR to ATR with aging, the change of PCA magnitude and axis orientation may be some kind of adaptation mechanism to the change of ACA. However, this compensation effect may be decreased with aging since the change of ACA is far greater than the PCA^28^.

Our study has some limitations. Firstly, the value of our findings and the impact of the PCA on corneal refractive surgery need to be further investigated, since the most relevant measurement for the corneal laser surgery is the manifest refraction and astigmatism. Moreover, the conclusions of our study can only be applied to myopic patients from the same age group. In older patients, the distribution and compensation effect of PCA may be different. Secondly, there are discrepancies between results for the posterior cornea using different technologies and devices^26,29^. Thus, our results obtained by the Pentacam might not be used interchangeably with PCA obtained from other devices. Thirdly, we are still unable to make accurate PCA estimation according to ACA measurements based on the results of our study. PCA measurement still relies on corneal tomographic instruments such as Pentacam, which is not always available in every ophthalmic setting. Fourthly, we did not investigate the change of PCA after corneal refractive surgery due to the cross-sectional design of the study. Further studies are needed to investigate this issue so that a sustainable astigmatism correction can be provided to the patients.

In conclusion, PCA is present in myopic patients having corneal refractive surgery and the PCA magnitude is increased with higher MA and with-the-rule ACA. Consideration of the impact of the PCA on laser astigmatism correction may be necessary.

## Methods

### Participants

This is a multicenter study involving five ophthalmic centers, including GZ (113.2° E 23.1° N, altitude 43.4 m), SY (123.4°E 41.8°N, altitude 51.0 m), CD (104.0° E 30.7° N, altitude 505.9 m), WH (114.2°E 30.4°N, altitude 23.3 m), and HK (114.1° E 30.4° N, altitude 27.6 m). The study has been approved by the Institutional Review Board (IRB) of Guangzhou Aier Eye Hospital and is in agreement with the Declaration of Helsinki. The same IRB also waived the need for informed consent since only review of medical records was conducted and no individual patient could be identified from the data. Retrospectively, digital medical records of patients that had preoperative assessment of corneal refractive surgery for myopia between 2017 and 2018 were reviewed and eyes meeting inclusion criteria were included consecutively. A total of 7829 eyes were included in the study (2227 eyes from GZ, 2434 eyes from SY, 1470 eyes from CD, 1431 eyes from WH, 267 eyes and from HK). Inclusion criteria were myopic eyes with an SE ≤ -0.50 D and good quality Scheimpflug scans, a stable refractive error (≤ 0.50 D of refractive error change in the past 2 years). We only included the right eye of each patient for analysis. Exclusion criteria included coexisting corneal diseases, keratoconus, forme fruste keratoconus, severe dry eye, previous ocular trauma or surgery, uveitis, glaucoma, wearing contact lenses within the previous 2 weeks, age younger than 18 years (unstable refraction) or older than 40 years (to reduce the astigmatism caused by the crystal lens).

### Examinations

All the eyes underwent routine preoperative examinations including best-corrected visual acuity (BCVA), intraocular pressure (IOP), cycloplegic and manifest refraction, anterior segment examination by slit-lamp, corneal topography and tomography measurements. The eyes were divided into three groups according to the manifest SE: low myopia (− 3.00 D < SE ≤  − 0.50 D), moderate myopia (− 6.00 D < SE ≤  − 3.00 D) and high myopia (SE ≤  − 6.00 D). The eyes were also divided into five groups according to the MA: MA < 0.50 D, 0.50 D ≤ MA < 1.00 D, 1.00 D ≤ MA < 2.00 D, 2.00 D ≤ MA < 3.00 D, MA ≥ 3.00 D.

Pentacam examination was performed by experienced technicians. The Pentacam instrument (Pentacam HR, Oculus GmbH, Wetzlar, Germany) was calibrated regularly on a weekly basis. The patients were positioned in front of the Pentacam instrument with the forehead and chin properly supported and both lateral canthi aligned with the marks. The patients were asked to blink 2–3 times to have the tear film evenly distributed on the cornea and then open the eyes wildly to stare at the fixation target while the instrument was proceeded to the cornea. Once the red cross on the screen coincided the red circle at the pupil center, the instrument automatically captured 50 rotational Scheimpflug images within 2 s. The power and axis of the keratometric astigmatism and PCA were measured within the central 3 mm using a default refractive indexes for the cornea (1.376) and aqueous humor (1.336). The procedure was performed again if the patient’s eye blinked during the measurement or quality of the scan was poor (comment on the display marked yellow or red). Only image covered at least central 8.0 mm of corneal surface and image quality labelled with ‘OK’ on the display was accepted. Pentacam data of the eyes were retrieved from the machine and only results with image quality labelled with ‘OK’ were included.

ACA was defined as WTR when the steepest meridian was 90° ± 30°, as ATR when the steepest meridian was between 0–30° or 150–180°, and as OBL when the steepest meridian > 30° and < 60°, or > 120° and < 150°. Because the posterior corneal surface has negative refractive power (divergent), we used other classification labels for the PCA to avoid confusion with the ACA. The PCA was defined as SMV when the steepest meridian was 90° ± 30°, as SMH when the steepest meridian was between 0°–30° and 150°–180°, and as SMO in the rest. Aggregate PCA was calculated as previously described^30^.

### Statistical analysis

The data from the five ophthalmic centers were pooled together for analysis. A Kolmogorov–Smirnov (KS) test was used to evaluate normality of all variables. Data of PCA, age, and SE were presented as mean ± standard deviation (SD). Kruskal–Wallis test was used for comparison of PCA, SE, age, and sex distribution among different groups. Dunn’s test was used for post hoc analysis. Spearman correlation test was used for correlation analysis between two groups of data. *P* < 0.05 was considered to be statistically significant.

## Data Availability

The data used during the current study are available from the corresponding author on reasonable request.
